# Quantifying natural seasonal variation in mutation parameters with mutation accumulation lines

**DOI:** 10.1002/ece3.4085

**Published:** 2018-05-02

**Authors:** Matthew T. Rutter, Angela J. Roles, Charles B. Fenster

**Affiliations:** ^1^ Department of Biology College of Charleston Charleston South Carolina; ^2^ Department of Biology Oberlin College Oberlin Ohio; ^3^ Department of Biology and Microbiology South Dakota State University Brookings South Dakota

**Keywords:** *Arabidopsis thaliana*, environmental variance, fitness, mutation, mutational variance, standing genetic variation

## Abstract

Mutations create novel genetic variants, but their contribution to variation in fitness and other phenotypes may depend on environmental conditions. Furthermore, natural environments may be highly heterogeneous. We assessed phenotypes associated with survival and reproductive success in over 30,000 plants representing 100 mutation accumulation lines of *Arabidopsis thaliana* across four temporal environments at a single field site. In each of the four assays, environmental variance was substantially larger than mutational variance. For some traits, whether mutational variance was significantly varied between seasons. The founder genotype had mean trait values near the mean of the distribution of the mutation accumulation lines in all field experiments. New mutations also contributed more phenotypic variation than would be predicted, given phenotypic and sequence‐level divergence among natural populations of *A. thaliana*. The combination of large environmental variance with a mean effect of mutation near zero suggests that mutations could contribute substantially to standing genetic variation.

## 1 INTRODUCTION

Mutations are the ultimate source of genetic variation; consequently, all biodiversity reflects the input of mutation to the evolutionary process (Nei, 2013). The mutation rate and the distribution of fitness effects of mutation figure prominently in evolutionary theory in such varied subjects as adaptation (e.g., Fisher, 1930; Orr, 1998), the evolution of sex (e.g., Kondrashov, 1988; Muller, 1964), and expectations about standing genetic variation (Barrett & Schluter, 2008; Haldane, 1937). The effects of mutation may also depend on environmental conditions. Whether a mutation is neutral, beneficial, or deleterious can vary across spatial and temporal environments or with environmental quality (Hietpas, Bank, Jensen, & Bolon, 2013; Kraemer, Morgan, Ness, Keightley, & Colegrave, 2016; Latta et al., 2015; Martin & Lenormand, 2006, 2015). Unfortunately, except for a very few studies (Roles & Conner, 2008; Roles, Rutter, Dworkin, Fenster, & Conner, 2016; Rutter, Shaw, & Fenster, 2010; Rutter et al., 2012) we have a relatively poor understanding of the effect of mutation on fitness under natural conditions and thus few direct measures of the temporal and spatial variability in mutation rates and effects outside of the laboratory.

Mutation accumulation lines (MA lines) have been used for 50 years to quantify mutation parameters (reviewed in Halligan & Keightley, 2009). MA lines are inbred lines which themselves are derived from inbred founders; thus, any genetic differences among the lines represent the fixation of spontaneous mutations in the different lines. The lines are maintained by a small sample of progeny each generation, and in the case of selfing organisms, for example, *Arabidopsis thaliana*, they are maintained with the minimum effective population size of one. Consequently, there is minimal bias in the fixation of deleterious versus beneficial mutations within the lines (Lynch & Walsh, 1998). The amount of among‐line variance due to genetic sources can be used to estimate mutation rate for a phenotype, and by assaying the premutation founder simultaneously with the MA lines, one can estimate the distribution of mutation effects, and whether they increase or decrease the value relative to the founder phenotype (e.g., Bataillon, 2000; Heilbron, Toll‐Riera, Kojadinovic, & MacLean, 2014; Lynch et al., 1999; MacKenzie, Saadé, Le, Bureau, & Schoen, 2005; Morgan, Ness, Keightley, & Colegrave, 2014; Rutter et al., 2010; Shaw, Geyer, & Shaw, 2002). It is typically assumed that the vast majority of mutants that affect fitness will decrease fitness relative to the founder (Keightley & Lynch, 2003).

To date, most studies utilizing the MA line approach have been conducted under controlled or laboratory conditions. Despite the elegance of these laboratory studies, many mutations with fitness consequences may be cryptic, when factors that typically deleteriously affect an organism, such as disease or predation, are minimized in controlled conditions. Controlled experiments also imply that environmental conditions are uniform and their effects are also minimized, perhaps leading to overestimates of mutation effects on the phenotype relative to environmental effects. Controlled experiments will also fail to detect season‐to‐season or site‐to‐site variation as sources of environmental effects that influence mutation parameters. There is some evidence suggesting that season‐to‐season variation is an important component of balancing selection (Delph & Kelly, 2014; Schemske & Bierzychudek, 2001) and may be involved in the maintenance of genetic variation and the role of mutations thereof (Connallon & Clark, 2015; Haldane & Jayakar, 1963).

Previously mutation parameters had been quantified in one field and one greenhouse planting for 100 *A. thaliana* MA lines developed by Shaw et al. (2002) from a Columbia founder (Rutter et al., 2010). The fates of five of these same 100 MA lines were described from multiple field environments (Rutter et al., 2012). These five lines were also sequenced. Based on the MA line variance and performance relative to the founder, we quantified a high rate of beneficial mutations and a high genomic mutation rate for fitness (haploid mutation rate = 0.12). A large contribution of environment to phenotype resulted in a low estimate of the contribution of mutation to standing genetic variation for fitness ($h_{m}^{2}$ = 0.0001) (Rutter et al., 2010). A significant genotype × environment interaction was found for the five MA lines (Rutter et al., 2012). For 50 of these lines grown across two spatial environments, significant variance genotype by environment interactions was also found (Roles et al., 2016).

Here, we extend the previous findings of Rutter et al. (2010, 2012) using the same 100 *A. thaliana* MA lines, with phenotypes measured across a temporal environmental scale. We present the mutational variances and the distribution of mutation effects across four natural field environments, for fitness and fitness components. We ask: (1) Is the observation of many mutations that are not deleterious found consistently across replicated experiments? (2) How do mutation effects scale to environmental variation? We planted our experiments in two fall and spring seasons in a nearly identical location to test the effects of mutations. Our planting times correspond to the two life histories exhibited by *A. thaliana* in its native range: winter annual (fall planting) and spring ephemeral (spring planting). Here, we focus on variance G × E that is whether the mutation parameters having to do with genetic variation introduced by mutation vary among seasons.

## 2 METHODS

The 100 lines of *A. thaliana* used in these experiments were the products of 25 generations of mutation accumulation. Methods for generating the lines are described previously (Rutter et al., 2010, 2012; Shaw et al., 2002). Briefly, all lines were derived from a single progenitor of the Columbia accession (the progenitor individual differs from the sequenced Col‐0 line by a few mutations; Ossowski et al., 2010). Six sublines were created from the progenitor line and were kept in cold storage (4.5°C). Individual MA lines were propagated by choosing a single seed at random to found the next generation. This procedure minimizes the potential for selection for or against mutations during the accumulation period. The lines were propagated for 24 generations at the University of Minnesota and for a 25th generation at the University of Maryland.

After mutation accumulation, field sublines were created for all 100 MA lines, along with field sublines of each progenitor subline. All field sublines were grown simultaneously in a greenhouse at the University of Maryland. The field subline plants grown in this single event produced seed that were used in all subsequent field experiments. Multiple field sublines were used for each MA line and for the progenitor line to dilute the idiosyncrasies of environmental effects on seed production by a particular maternal plant. Thus, seed from all lines in an experiment was of the same age and from maternal plants grown in the same environment.

Field experiments were carried out in spring 2004, fall 2004, spring 2005, and fall 2005, with the following previously described methods (Rutter et al., 2010, 2012). Before each experiment, seeds were placed on moist potting soil in groups of 20 per germination pot, cold‐treated at 4°C, and moved to a greenhouse for germination. Ten days after removal from the cold, germination was assessed in each pot, and seedlings were transplanted from germination pots to a 144‐well flat. Positions within the flat corresponded to field positions, and thus, plants from the 100 MA lines and the progenitor line were randomized within each of 14 blocks, where each block used plants from four flats (three filled flats and one partially filled flat). Within each block, each field subline was represented by one to three plants, for a total of five plants from each MA line and 36 plants from the progenitor (six plants for each of the six progenitor lines), yielding 500 MA plants and 36 progenitor plants per block. Three to five field sublines represented each MA line, and six field sublines were used for each of the six progenitor sublines. However, due to variability in seed production across maternal plants, for the MA lines, field subline identity was not perfectly identical across experiments, although multiple field sublines represented each MA line in every experiment. This set of 7,504 plants (7,000 MA plants and 504 progenitor plants) was used as a basic framework across all four field experiments. As described below, some additional Col‐0 plants were also grown in two experiments.

At approximately 15 days after plants had been removed from the cold, they were transplanted into a field site at Blandy Experimental Farm, located at Boyce, Virginia, USA (39°03′45.1″N, 78°03′30.5″W). At this point, seedlings typically had two to four true leaves and were robust to transplant shock. To simulate the early successional habitat of *A. thaliana*, the field sites were treated with herbicide 3 months before planting and were plowed 3–6 weeks before transplant. There was little vegetation at the time of transplant, but local vegetation became established in the plots during each of the experiments.

Seedlings were transported from the greenhouse to the field site in flats and then were transplanted along with their soil plug into the field site. Plants within a flat were planted in a rectangular grid with plants spaced 10 cm apart from each other. Flats in the same block were separated by 1 m within each block. Blocks were separated spatially from each other by 2 m. The plowed area extended several meters beyond the experimental area. Plants that died during the first 3 days of transplant (likely from transplant shock) were replaced with other plants from the same MA line. Plants were censused regularly for survival. When roughly 50% of the plants had flowered, we performed a census of whether each plant was flowering (the census did not occur in the fall 2005 experiment). For both fall and spring plantings, all plants had senesced and were harvested in May. Biomass, counts of total full and aborted fruits (measuring less than 5 mm) and a sample of fruit size were taken from dried plant material from each plant. The fall 2004 experiment was an exception in which less data were collected, as described below. We report analyses on these measures as well as on a cumulative fitness measure that combines survival and full fruit produced by counting any plant that did not survive to reproduce as having zero fitness.

Seedlings were planted in spring and fall, reflecting the two life histories of *Arabidopsis*, and the plantings occurred in each of 2 years (2004 and 2005). The founder Columbia line is capable of acting as a spring ephemeral and as a winter annual, but the typical life history of the source population of Columbia is unknown. In both life histories, plants senesce at the field site with similar timing, in late April or early May. Although the independent plantings differed temporally, they were spatially clustered within the same 200 m^2^ area at Blandy Experimental Farm.

It is possible that the premutation founder Columbia individuals have declined in fitness relative to the sequenced line commonly used in the study of *Arabidopsis*; this could explain why the distribution of mutational effects includes fewer harmful mutations. To test whether the founder line is underperforming Columbia, additional 140 seeds from the sequenced Columbia genotype were obtained directly from the Arabidopsis Biological Research Center. Five plants from this genotype were included in each block in the spring and fall 2005 plantings.

In the fall 2004 experiment, the plants were very large and overall fruit numbers were extremely high. In this case, we found that fruit number was highly correlated with biomass (*r* ^2^ = .96) in a randomly selected subset of the plants (Rutter et al., 2012) and we thus estimated fruit number from biomass for the fall 2004 planting rather than measuring fruit number directly.

### 2.1 Statistical analysis

In this study, mutational variance is estimated by the among‐line variance of the MA lines. We used a mixed‐modeling approach to estimate and test the significance of mutational variance. Due to unequal sampling of progenitor lines and MA lines, we performed univariate analyses to estimate variance on the MA lines alone. Similarly, we analyzed each experiment separately. All analyses were performed in R version 3.3.3 (R Core Team, 2017). The response variables consist of counts, continuous measurements, or binary responses. Count variables include number of flowers, number of aborted fruits, number of good fruits, and total fitness (the product of germination, survival, and number of fruits). Continuous measurements include biomass and fruit length. Binary variables include germination, survival, flowering, and percent aborted fruits. Germination includes all seeds planted in the greenhouse prior to transplantation to the field. Survival and fitness include all individuals planted in the field. All other variables are subsets that include only individuals that survived to flowering. For count and binary variables, we used the appropriate distribution (Poisson and binomial, respectively) in our analyses. We analyzed each response variable using (1) likelihood‐based estimation with the lme4 library (version 1.1‐13; Bates, Maechler, Bolker, & Walker, 2015) and (2) Bayesian Markov chain Monte Carlo generalized linear mixed models with the MCMCglmm library (version 2.24; Hadfield, 2010).

The full model for a given experiment included the random effect of block, the random effect of line, the random effect of field subline nested within line, and residual error. For each response variable, we evaluated which explanatory variables to include in the final MCMCglmm model with a parametric bootstrap using the maximum‐likelihood fit; this approach takes advantage of the robust estimates from MCMCglmm while accounting for the instability of such models when using a prior for random effects with low variance. Model fitting of effects allowed us to (1) evaluate the importance of covariates (such as field subline or block) and (2) identify and address instability introduced by some explanatory variables. First, we constructed a series of glmer model pairs with one model including and the other excluding an explanatory variable and we calculated the observed likelihood ratio of the deviance for each model pair. Next, we ran 1,000 replicates of the parametric bootstrap, fitting the simulated data to the model pair and constructing the likelihood ratio of the deviance for each replicate. Finally, we calculated the probability of our observed likelihood ratio as the fraction of replicates in which the simulated likelihood ratio was larger than the observed likelihood ratio. If this *p*‐value was below .05, then we considered the focal variable to have significant explanatory power and retained the variable for the MCMCglmm analysis. Having determined the explanatory variables to retain in the model for each response variable, we then ran that model using MCMCglmm and used the results to estimate our parameters of interest. We used MCMCglmm parameter estimates because these models produce more robust estimates of the random effects for non‐Gaussian data (Hadfield, 2010).

For each response variable, the per‐generation increase in genetic variance due to mutation (mutational variance, *V*_m_) was calculated as $V_{m}=\frac{V_{l}}{2t}$, where *V*_l_ is the among‐line variance and *t* is the number of generations of divergence (Lynch & Walsh, 1998). Mutational heritability ($h_{m}^{2}$), which is the rate of increase in heritability due to new mutations, was calculated as $h_{m}^{2}=\frac{V_{m}}{V_{e}}$ (Houle, Morikawa, & Lynch, 1996). We also calculated the mutational coefficient of genetic variation CV_m_, which is scaled to the mean by computing 100($\frac{\sqrt{V_{m}}}{\overset{¯}{x}}$) where $\overset{¯}{x}$ is the trait mean for Gaussian response variables (Lande, 1975; Lynch & Walsh, 1998). For Poisson variables, CV_m_ was calculated as $100(e^{\sqrt{V_{m}}}-1)$ for ln scale. For each of these estimates, 95% CIs were estimated from the posterior distributions of the MCMCglmm output. Note that non‐normality of the data and the use of log or logit transformations in analysis will influence the estimates. For example, the transformations eliminate the multiplicativity of the fitness components estimated from the model outputs.

To compare mean trait values for the overall set of MA lines with the founder and Columbia from the stock center, a series of paired *t* tests were conducted for each trait within each experimental planting and for a combined dataset that included all four experimental plantings. *T* test comparisons were between the MA line means and the founder, MA lines and Columbia, and Columbia and the founder. *T* tests involving Columbia were restricted to the two plantings for which this accession was included.

## 3 RESULTS

The four planting seasons differed substantially in plant performance. In both spring seasons, adult reproductive plants were small. Mortality varied considerably between the two spring plantings, with higher prereproduction mortality of seedlings transplanted in spring 2005 (39%, *SE*: 0.56%) compared with spring 2004 (21%, *SE*: 0.47%), possibly because the conditions during the spring growing season were cooler and drier in 2005 (Table S1). In both fall seasons, mortality over the winter was high, with higher mortality in the winter of 2005–2006 (73%, *SE*: 0.51%) than in 2004–2005 (59%, *SE*: 0.57%). Again, conditions in December 2005 (during rosette development) were cooler and drier than in 2004. Those plants that successfully survived the winter had much higher fruit production than plants transplanted in the spring. Overall, average fitness of the MA lines was highest in fall 2004–2005 and lowest in fall 2005–2006. The spring plantings had intermediate fitness or fitness close to fall 2005–2006, due to the combination of lower mortality and smaller size. Germination occurred in the greenhouse prior to transplantation, and there was little variation in overall germination rate (spring 2004: 93%; fall 2004: 96%; spring 2005: 96%; fall 2005: 94%).

In the context of the seasonal variation in environmental conditions, some mutational parameters changed across seasons while others remained consistent. Mutational variance among the MA lines was significant in at least one season for every trait measured from plants grown in the field except fruit length (Table 1). For four traits (the number of aborted fruits, the number of filled fruits, the number of flowers, and the integrated fitness measure), significant mutational variation was found in every season. For biomass, the timing of flowering, and survivorship, significant mutational variance was found in some seasons but not others.

**Table 1:** *p*‐values for among‐line variance for 100 MA lines derived from the Columbia accession of *Arabidopsis thaliana* for each trait in each experiment

Trait	Spring 2004	Spring 2005	Fall 2004	Fall 2005
Survival	**.002**	.083	.106	**.018**
Timing of flowering	.159	**.004**	**.001**	
Biomass	.415	.065	**.002**	.294
Number of flowers	**.001**	**.001**		**.001**
Fruit length	.421	.820		.083
Fruit number	**.001**	**.001**		**.001**
Number of aborted fruits	**.001**	**.001**		**.001**
Fitness estimate	**.001**	**.001**	**.001**	**.001**

Missing cells represent traits that were not measured.

Significant variances are in bold.

Environmental variances were generally higher in fall plantings than in spring (mortality was high and surviving plants were large in the fall planting experiment), except for the number of aborted fruits, which had similar environmental variances in the spring of 2004 and fall of 2005 (Table 2). Environmental variances for overall fitness were especially high for the fall plantings relative to the spring plantings. Despite the differences in environmental variance, measures of $h_{m}^{2}$ were in a narrow range between 0.0001 and 0.0004, with a somewhat higher estimate of $h_{m}^{2}$ for fruit length, 0.0006, in the fall 2005 planting. While mean trait values differed between seasons for traits measured in the field, there was no clear consequence for $h_{m}^{2}$ values. Most of the measures of the coefficient of mutational variance also showed little season‐to‐season variability.

**Table 2:** MCMCglmm parameter estimates for measured traits, with confidence intervals for 100 MA lines derived from the *Columbia* accession of *Arabidopsis thaliana*

Experiment	*V*_l_	Mean	*V*_m_	$h_{m}^{2}$
Germination				
Spring 2004	0.4578 (0.2933–0.6319)	3.2069 (3.0598–3.3563)	0.0092 (0.0059–0.0126)	0.0092 (0.0059–0.0126)
Spring 2005	0.4476 (0.28–0.6248)	3.5693 (3.4128–3.7384)	0.009 (0.0056–0.0125)	0.009 (0.0056–0.0125)
Fall 2004	0.3544 (0.2332–0.4895)	3.358 (3.2176–3.4875)	0.0071 (0.0047–0.0098)	0.0071 (0.0047–0.0098)
Fall 2005	0.2476 (0.1459–0.3638)	2.9594 (2.8473–3.0899)	0.005 (0.0029–0.0073)	0.005 (0.0029–0.0073)
Survival				
Spring 2004	0.0559 (0.0053–0.1095)	1.6249 (1.3668–1.9022)	0.0011 (1e‐04‐0.0022)	0.0011 (1e‐04‐0.0022)
Spring 2005	0.0145 (2e‐04‐0.0399)	0.5695 (0.3367–0.8054)	3e‐04 (0‐8e‐04)	3e‐04 (0‐8e‐04)
Fall 2004	0.0138 (2e‐04‐0.0381)	−0.1876 (−0.6521 to 0.2573)	3e‐04 (0‐8e‐04)	3e‐04 (0‐8e‐04)
Fall 2005	0.0403 (3e‐04‐0.0948)	−2.2905 (−2.756 to 1.786)	8e‐04 (0–0.0019)	8e‐04 (0–0.0019)
Flowering timing				
Spring 2004	0.0205 (2e‐04‐0.066)	2.2734 (1.9722–2.6019)	4e‐04 (0–0.0013)	4e‐04 (0–0.0013)
Spring 2005	0.0382 (9e‐04‐0.0729)	−0.0861 (−0.3019 to 0.1087)	8e‐04 (0–0.0015)	8e‐04 (0–0.0015)
Fall 2004	0.038 (6e‐04‐0.0733)	−0.0883 (−0.2837 to 0.124)	8e‐04 (0–0.0015)	8e‐04 (0–0.0015)

Experiment	*V*_l_	*V*_e_	Mean	*V*_m_	$h_{m}^{2}$	CV_m_
Biomass						
Spring 2004	0	0.0051 (0.0049–0.0052)	0.0764 (0.0629–0.0891)	0	0	0.4534 (3e‐04‐0.986)
Spring 2005	1e‐04 (0‐2e‐04)	0.0127 (0.0121–0.0132)	0.1152 (0.0856–0.1439)	0	1e‐04 (0‐3e‐04)	0.9527 (0.0021–1.6749)
Fall 2004	0.0188 (0–0.0348)	1.2465 (1.1852–1.3082)	1.5441 (1.3178–1.7939)	4e‐04 (0‐7e‐04)	3e‐04 (0‐6e‐04)	1.2215 (0.565–1.9191)
Fall 2005	0.0057 (0–0.0173)	0.2754 (0.2433–0.3084)	0.4807 (0.3595–0.5942)	1e‐04 (0‐3e‐04)	4e‐04 (0–0.0013)	1.9336 (0.001–4.0822)
Flower number						
Spring 2004	0.0064 (0–0.0124)	0.8503 (0.8156–0.8843)	3.863 (3.6364–4.0796)	1e‐04 (0‐2e‐04)	1e‐04 (0‐3e‐04)	37.1881 (36.9203–37.4021)
Spring 2005	0.0023 (0–0.0066)	0.6943 (0.6637–0.7269)	4.1752 (3.939–4.3935)	0 (0‐1e‐04)	1e‐04 (0‐2e‐04)	37.0043 (36.7879–37.2117)
Fall 2005	0.0154 (0–0.0522)	0.9911 (0.8732–1.1098)	5.7975 (5.5592–6.0453)	3e‐04 (0–0.001)	3e‐04 (0–0.0011)	37.3338 (36.7882–37.996)
Fruit number						
Spring 2004	0.0189 (0–0.036)	2.1006 (2.0044–2.209)	2.2052 (1.8322–2.5964)	4e‐04 (0‐7e‐04)	2e‐04 (0‐3e‐04)	37.4831 (37.1004–37.8759)
Spring 2005	0.0049 (0–0.0147)	1.6457 (1.5604–1.7267)	3.122 (2.7842–3.4504)	1e‐04 (0‐3e‐04)	1e‐04 (0‐2e‐04)	37.0997 (36.788–37.4242)
Fall 2005	0.0405 (0–0.1322)	2.5413 (2.2097–2.8685)	4.1872 (3.6256–4.6538)	8e‐04 (0–0.0026)	3e‐04 (0–0.0011)	37.6815 (36.788–38.7289)
Fruit length						
Spring 2004	0.009 (0–0.0291)	5.0103 (4.8091–5.2004)	10.448 (10.2285–10.6593)	2e‐04 (0‐6e‐04)	0 (0‐1e‐04)	0.1074 (0–0.2312)
Spring 2005	0.0109 (0–0.0388)	7.7217 (7.3919–8.0532)	11.4007 (10.996–11.7689)	2e‐04 (0‐8e‐04)	0 (0‐1e‐04)	0.1062 (1e‐04‐0.2452)
Fall 2005	0.1408 (0–0.3921)	4.8451 (4.2725–5.4585)	10.1248 (9.6014–10.6006)	0.0028 (0–0.0078)	6e‐04 (0–0.0017)	0.458 (0.0014–0.8763)
Number of fruit aborted						
Spring 2004	0.0043 (0–0.0097)	0.8266 (0.7938–0.8612)	3.5035 (3.312–3.6675)	1e‐04 (0‐2e‐04)	1e‐04 (0‐2e‐04)	37.1073 (36.8193–37.3279)
Spring 2005	0.0018 (0–0.0056)	0.6281 (0.5985–0.6572)	3.5235 (3.306–3.7272)	0 (0‐1e‐04)	1e‐04 (0‐2e‐04)	36.9802 (36.788–37.1782)
Fall 2005	0.0115 (0–0.0384)	0.8174 (0.7206–0.9169)	5.4068 (5.1738–5.646)	2e‐04 (0‐8e‐04)	3e‐04 (0–0.001)	37.2578 (36.7884–37.8216)

Experiment	*V*_l_	Mean	*V*_m_	$h_{m}^{2}$		
Percent of fruit aborted						
Spring 2004	0.0317 (0.0228–0.0415)	−1.0723 (−1.3105 to −0.8314)	6e‐04 (5e‐04‐8e‐04)	6e‐04 (5e‐04‐8e‐04)		
	
Spring 2005	0.0339 (0.0244–0.0439)	−0.0871 (−0.2993 to 0.1091)	7e‐04 (5e‐04‐9e‐04)	7e‐04 (5e‐04‐9e‐04)		
Fall 2005	0.262 (0.1901–0.3381)	−0.9293 (−1.3637 to −0.5135)	0.0052 (0.0038–0.0068)	0.0052 (0.0038–0.0068)		
	

Experiment	*V*_l_	*V*_e_	Mean	*V*_m_	$h_{m}^{2}$	CV_m_
Fitness						
Spring 2004	0.0457 (0.014–0.0851)	4.6265 (4.3953–4.8577)	1.3148 (0.8884–1.7533)	9e‐04 (3e‐04‐0.0017)	2e‐04 (1e‐04‐4e‐04)	37.8942 (37.4358–38.3599)
Spring 2005	0.0305 (0–0.079)	9.4795 (8.9818–9.9738)	0.9133 (0.3806–1.4373)	6e‐04 (0–0.0016)	1e‐04 (0‐2e‐04)	37.6109 (36.789–38.2796)
Fall 2004	0.1202 (0–0.3306)	41.0072 (38.822–43.3942)	−0.0902 (−1.3618 to 1.1326)	0.0024 (0–0.0066)	1e‐04 (0‐2e‐04)	38.4165 (36.7886–39.9042)
Fall 2005	1.5719 (0–3.2297)	91.9985 (79.3242–106.0156)	−14.7184 (−17.1283 to −12.3724)	0.0314 (0–0.0646)	3e‐04 (0‐7e‐04)	43.5537 (37.5226–47.8996)

**Table 3:** Untransformed means and 95% confidence intervals for the MA lines, founder lines, and Col‐0 lines for all traits and experiments

Trait	Experiment	MA mean	Founder mean	Col‐0 mean
Percent surviving to reproduction	Spring 2004	78.9 (77.9–79.8)	78.2 (74.6–81.8)	
Percent surviving to reproduction	Fall 2004	45.5 (45.3–47.7)	43.8 (39.5–48.1)	
Percent surviving to reproduction	Spring 2005	61.3 (60.1–62.4)	61.5 (57.2–65.8)	21.4 (15.8–27)
Percent surviving to reproduction	Fall 2005	14.4 (13.5–15.2)	16.5 (13.2–19.7)	11 (6.6–15.2)
Biomass	Spring 2004	0.0769 (0.075–0.0789)	0.079 (0.0714–0.0866)	
Biomass	Fall 2004	1.48 (1.44–1.52)	1.42 (1.27–1.57)	
Biomass	Spring 2005	0.119 (0.116–0.123)		0.112 (0.075–0.149)
Biomass	Fall 2005	0.50 (0.46–0.55)	0.48 (0.34–0.63)	0.57 (0.12–1.03)
Number of fruits	Spring 2004	22.1 (21.4–22.8)	22.7 (19.9–25.5)	
Number of fruits	Spring 2005	48.2 (46.5–49.8)	46.1 (40–52.2)	48.2 (30.4–65.9)
Number of fruits	Fall 2005	198 (178–218)	196 (126–265)	223 (41–405)
Number of aborted fruits	Spring 2004	49.7 (48.5–50.9)	50.7 (46–55.3)	
Number of aborted fruits	Spring 2005	48.4 (47.2–49.6)	47.5 (42.9–52.1)	43.5 (33.1–53.9)
Number of aborted fruits	Fall 2005	321 (299–343)	320 (244–397)	276 (121–432)
Percent fruit aborted	Spring 2004	73.4 (72.9–74)	72.9 (70.8–75)	
Percent fruit aborted	Spring 2005	56.8 (56.1–57.5)	57 (54.3–60)	60.6 (53.3–67.9)
Percent fruit aborted	Fall 2005	69.2 (67.5–70.9)	71.1 (65.2–77)	60.5 (44–76.9)
Fruit length	Spring 2004	10.5 (10.4–10.5)	10.4 (10.1–10.7)	
Fruit length	Spring 2005	11.5 (11.4–11.6)	11.2 (10.9–11.6)	11.2 (10.3–12.2)
Fruit length	Fall 2005	10.2 (10–10.4)	9.8 (9.1–10.5)	11.1 (9.4–12.7)
Fitness	Spring 2004	17.4 (16.8–18)	17.7 (15.3–20.1)	
Fitness	Fall 2004	357 (344–370)	325 (281–369)	
Fitness	Spring 2005	29.5 (28.4–30.7)	28.4 (24.1–32.6)	10.3 (5.7–14.9)
Fitness	Fall 2005	17.1 (14.9–19.2)	19.0 (10.7–27.3)	11.7 (1.1–22.2)

The mean trait value for the MA lines did not differ significantly from the founder for any trait, either in single experiments or across all experiments (all *p*‐values >.15) (Table 3). Plants grown from Columbia accession seed provided by the stock center and grown in the spring 2005 planting had significantly lower survival (*p* < .0001) and total fitness (square root‐transformed, *p* < .0001) than the MA plants and founder plants. In the fall 2005 planting, there were no significant differences for any trait between the plants grown from stock center and the MA and founder plants.

## 4 DISCUSSION

### 4.1 Distribution of mutational effects

There was never a significant difference between founder trait values and the overall mean trait values of the MA lines in any trait in any season. Similar means between the founder and mutant lines are not surprising for the cases for which there was no detectable mutational variance. However, when mutational variance was present, we still found no evidence of a decline in overall mean fitness. These findings were true for traits that are direct components of fitness, such as fruit production and survival. Similar results have been found previously for these mutation accumulation lines (Roles et al., 2016; Rutter et al., 2010; Shaw et al., 2002) and independently derived MA lines from the same founder (MacKenzie et al., 2005). In total, these studies represent a collection of nine assays of lines derived from this founder: six in the field (across two sites and four seasons) and three in greenhouse conditions. Clearly, the similar performance of MA lines and founders is not an idiosyncrasy of a single assay. As another measure of wild‐type performance, Columbia lines that were obtained directly from the Arabidopsis stock center actually had lower trait values for fitness in the field than either the founder line or the MA lines, primarily due to lower survivorship. However, because these plants originated from seed directly provided by the Arabidopsis Biological Resource Center, phenotypic differences might reflect effects of seed age or the seed production environment. The Columbia seeds representing the founder, on the other hand, were generated at the same time as the MA line seeds. In addition, although the founder was generated from the Columbia accession, it does carry several mutations that differ from the Columbia reference (Ossowski et al., 2010). Our findings are an exception to the widely held assumption that nearly all mutations are deleterious (Bataillon, 2003; Keightley & Lynch, 2003). There are other reports of high frequencies of beneficial mutations that are either induced or spontaneous (Hall, Mahmoudizad, Hurd, & Joseph, 2008; Perfeito, Fernandes, Mota, & Gordo, 2007; Schaack, Allen, Latta, Morgan, & Lynch, 2013; Zhang, Azad, & Woodruff, 2011).

Given that our founder genotype originates from Northern Germany and has experienced a series of inbreeding events throughout its laboratory cultivation, and our experiments were conducted in the novel environment of the Virginia Blue Ridge, our observation of a lack of an overall deleterious effect of mutation may be consistent with Fisher's (1930) geometric model. In this case, the assayed population may be far from its optimum in the new environment, increasing the likelihood a mutation will be beneficial, as has been observed with experimental studies of microorganisms (Burch & Chao, 1999; Khan, Dinh, Schneider, Lenski, & Cooper, 2011; Kryazhimskiy, Rice, Jerison, & Desai, 2014; MacLean, Perron, & Gardner, 2010; Perfeito, Sousa, Bataillon, & Gordo, 2014) and in one field study with *A. thaliana* (Stearns & Fenster, 2016). It is also possible that there was within‐plant selection during the propagation of the mutation accumulation lines (Otto & Orive, 1995). However, new explanations may be required to describe the conditions that lead to the phenomenon of a symmetric distribution of mutational effects.

### 4.2 Variance G × E

Given that the MA lines shared nearly identical sets of mutations across all of the assay environments, differences in MA line variance are likely due to the different expression of mutations in each assay environment (Latta et al., 2015); for example, epigenetic differences between assays or between MA lines may explain some MA line differences (Jiang et al., 2014). As mutational variance approaches zero, there is decreasing potential for selection to act on the new mutations. Even mutations that are deleterious in some environments are likely to be neutral in environments with very small mutational variance. If environments in which mutations have little effect are common, standing genetic variation could be maintained even when there is strong phenotypic selection.

The contrasting contribution of mutations and environment to phenotypic variation for any of the traits in our experiment, including fitness, is striking. The environmental effects typically had three to four orders of magnitude greater effect on producing variation than mutation. In this context, mutation effects are very small and mutations are more likely to be maintained within the population as standing genetic variation (Charlesworth & Charlesworth, 2010).

Seasonal variation in environmental variances was much more evident for some traits than others. For example, for biomass, fruit number, the number of flowers, and the number of aborted fruit the environmental variance differed by over an order of magnitude across seasons and by several orders of magnitude in some cases. However, for fruit length, seasonal differences in environmental variance were much smaller. Such varied response across traits likely reflects differences in the environmental contribution to trait values. Fruit length may be little influenced by environmental quality, while total fruit number may depend substantially on the quality of the environment. Trait means may be a useful proxy for describing environmental quality (e.g., higher mean fruit number would be expected in a higher quality or low stress environment), but changes in trait means did not clearly match changes in $h_{m}^{2}$, average performance in MA lines or mutational variances. Our finding is consistent with other broad surveys that indicate that environmental stresses do not change the strength of selection on new mutations in a consistent fashion—that is, making new mutations more deleterious or more beneficial on average (Agrawal & Whitlock, 2010; Martin & Lenormand, 2006). Poor environments may or may not allow selection to discriminate among new mutations, although there is evidence that the most stressful environments may magnify mutation effects (Kraemer et al., 2016). Similarly, larger environmental variance may or may not prevent selection from acting on new mutations.

### 4.3 Calibration of mutational variance with standing genetic variation

At the sequence level, mutation rates have been documented in an increasing number of organisms (e.g., Denver et al., 2012; Sung et al., 2012) and are typically about 1 × 10^−8^ at the nucleotide level and about one new mutation at the gamete level. At the phenotype level, mutation rates vary from about 0.01 to 0.1 for each new gamete, with estimates of mutational heritability, $h_{m}^{2}$, ranging from 10^−4^ to 10^−3^ (Lynch et al., 1999). Comparing the mutational variance with the variance among populations in a species allows us to calibrate the amount of phenotypic variance generated each generation by mutation with an estimate of the total extant phenotypic variance generated over millions of years of evolution in a species (*A. thaliana* diverged from the closely related *Arabidopsis lyrata* ~10 million years ago) (Hu et al., 2011). In this study, nearly all of our estimates of the per‐generation contribution of mutations to heritable genetic variance scaled to environmental variation ($h_{m}^{2}$) are consistently around 10^−4^. If we scale up our estimates of *V*_g_ to 25 generations of MA, then mutations have contributed on the order of 2 × 10^−3^ *V*_g_ scaled to *V*_e_ for fitness. In contrast, studies of accessions grown in field‐like settings, or RILs generated from an extreme cross of Italian and Swedish *A. thaliana* populations, similarly measured for fitness or fitness proxies found *V*_g_ scaled to *V*_e_ is much higher, on the order of 0.05–0.1 (Ågren, Oakley, McKay, Lovell, & Schemske, 2013; Rutter & Fenster, 2007; Samis et al., 2012; Stearns & Fenster, 2016), as one might expect. However, to put this into the context of mutational contributions to fitness variance, after only 25 generations of MA, the 100 lines have diverged such that this population of 100 MA lines has on the order of 25‐fold less fitness variation than found in a survey of 21 worldwide *A. thaliana* accessions grown at a single site (Rutter & Fenster, 2007). Presumably, the extant genetic variation among populations is attributable to a wider sampling of evolutionary process, including mutation, drift, gene flow, and selection. Seen in this way, mutations may contribute significantly to standing genetic variation at the population level. Consistent with this finding, phenotypic variation in scutellar bristle number among *Drosophila melanogaster* MA lines (Mackay, Fry, Lyman, & Nuzhdin, 1994; Mackay, Lyman, & Jackson, 1992) was greater than observed in a worldwide survey of *D. melanogaster* populations (Capy, Pla, & David, 1993).

We can also scale the contribution of mutations to heritable genetic variation for fitness phenotypes to the amount of sequence variation found among our MA lines as well as among accessions. The amount of sequence variation among the five sequenced MA lines is roughly three orders of magnitude less than among 80 sequenced natural accessions when focused solely on the private mutations (21 mutations versus 22,000 private alleles) (Cao et al., 2011; Ossowski et al., 2010). Thus, the mutations in our study scale up to express heritable phenotypic variation about 40‐fold more than expected based on sequence differences alone (as above, phenotypic differences between accessions are about 25 times greater among accessions than MA lines). Explanations for this observation include selective removal of deleterious mutations in native environments or epigenetic differences among the MA lines (Becker et al., 2011). Notably, similar differences between the amount of genetic and phenotypic variation were also found in a selection experiment with maize (Durand et al., 2015).

## 5 CONCLUSION

Our replicated field studies consistently demonstrate that the mutation accumulation lines have significant variance for life history and fitness characters, but do not differ on average from the founder for these traits. Such a result suggests that there is not an inherent preponderance of deleterious mutations in the *A. thaliana* Columbia background. Furthermore, while we quantified a relatively high haploid whole genomic mutation rate of 0.12 for fitness in one of the plantings (Rutter et al., 2010), we consistently observe $h_{m}^{2}$ to be low, an order of magnitude lower than many studies conducted in the laboratory (Lynch & Walsh, 1998). The likely explanation is that the low $h_{m}^{2}$ quantified here reflects elevated *V*_e_ under field conditions. Relatively high mutation rates for fitness in the context of large inputs of *V*_e_ combined with a high frequency of beneficial mutations all suggest that mutations can contribute substantially to standing genetic variation for fitness.

## CONFLICT OF INTEREST

None declared.

## AUTHOR CONTRIBUTIONS

MTR and CBF designed and conducted the experiments. MTR and AJR performed statistical analyses. MTR, AJR, and CMF wrote the manuscript.
