# Supplementary material for: Quantifying natural seasonal variation in mutation parameters with mutation accumulation lines
Source: Ecol Evol. 2018 May 2;8(11):5575–85. doi: 10.1002/ece3.4085 (PMC6010865; doi:10.1002/ece3.4085)
Supplement: Supplementary file 1 [file ECE3-8-5575-s001.docx]

Supplemental table. Temperature (°C) and precipitation (cm) during growing seasons. Spring planting: March- May, fall planting: November-April.

Avg Low High Precip

March 2004: 46 19 78 3.88

April 2004: 54 30 89 8.92

May 2004: 69 35 89 7.09

November 2004: 48 28 73 4.72

December 2004: 38 12 62 4.50

January 2005: 35 6 69 2.44

February 2005: 38 17 62 1.73

March 2005: 40 17 68 7.04

April 2005: 55 30 86 3.81

May 2005: 59 32 89 4.57

November 2005: 48 21 77 8.03

December 2005: 33 12 59 1.42

January 2006: 41 21 68 4.64

February 2006: 36 21 64 1.37

March 2006: 45 24 82 0.13

April 2006: 56 32 80 4.01

May 2006: 61 35 93 4.22
